# The Research Defence Society

**Published:** 1908-06-27

**Authors:** 


### The Research Defence Society.

The inaugural meeting of this Society was held on June 19 at the House of the Royal Society of Medicine, 20 Hanover Square, under the presidency of the Earl of Cromer. A large gathering of members was present, including a good proportion of ladies, and the proceedings throughout were most enthusiastic. It was noticeable that the speakers one and all struck a note of sincerity and profound conviction, and this was as evident among the distinguished barristers, politicians, diplomatists, and other quite non-medical orators as among the more strictly vivisectionist experts. This intensity of feeling lent a driving force to the speeches, which might well have convinced the more amateur sections of the anti-vivisection party that the question is one in which pure reason rather than sentiment, carefully-sifted facts rather than hysterical denunciation, and a quite judicial attitude generally are essential. It might well have opened their eyes, had any of them been present, to the fact that men more earnest and far more eminent than any of the leaders of their own side have considered the subject without prejudice towards either party, and have decided energetically to support the Research Defence Society, and to oppose the anti-vivisection propaganda. The Report having been received and adopted, the President addressed the meeting in a speech worthy of his reputation and of the cause. Most noteworthy, perhaps, was his reiterated declaration that the primary aim of the Society is the establishment of the truth, and the entire truth, about experiments on animals as conducted in this country, the reasons why they are undertaken, and the results derived from them. Lord Cromer made some telling points in referring to the progress of tropical medicine and hygiene during his residence in Egypt; progress, much of which was helped and made possible, we may remark, by his own assistance and encouragement. We do not know in what light the anti-vivisectionists regard Lord Cromer and his lifework; but this we do feel sure of, that Englishmen all the world over see in him the personification and the highest expression of those qualities of integrity, ability, and singleness of purpose which our race most admires. To suppose that such a man would interest himself in the defence of a profession entirely unconnected with his whole previous career, except under the compelling stress of what he regards as a matter of great importance to the welfare of the State, is to suppose something so far-fetched and preposterous that it may well make the more reasonable among them reconsider their position. The first resolution put to the meeting was:—"That the Research Defence Society be, and is hereby constituted, to make generally known the facts about experiments on animals in this country and the regulations under which they are conducted; the immense importance of such experiments to the welfare of mankind; and the great saving of human life and health which is already due to them." This was proposed by Sir Thomas Barlow, who said that he represented the rank and file of the practising members of the medical profession in defending those more directly concerned with vivisection. It was clear that the seconder, Lord Robert Cecil, K.C., M.P., felt strongly the justice of the cause he advocated; and the resolution was carried unanimously. Among the subsequent speakers were the Right Hon. Walter Long, M.P., Dr. C. J. Martin, Mr. H. T. Butlin, and the Hon. Sydney Holland.

The Society has made a most auspicious start in every way, and now that it is fairly launched it may look forward with confidence to a prosperous and successful career.

